# Calmodulin-binding transcription factor shapes the male courtship song in *Drosophila*

**DOI:** 10.1371/journal.pgen.1008309

**Published:** 2019-07-25

**Authors:** Kosei Sato, Md. Tanveer Ahsan, Manabu Ote, Masayuki Koganezawa, Daisuke Yamamoto

**Affiliations:** 1 Neuro-Network Evolution Project, Advanced ICT Research Institute, National Institute of Information and Communications Technology, Kobe, Japan; 2 Division of Neurogenetics, Tohoku University Graduate School of Life Sciences, Sendai, Japan; University of California Davis, UNITED STATES

## Abstract

Males of the *Drosophila melanogaster* mutant *croaker* (*cro*) generate a polycyclic pulse song dissimilar to the monocyclic songs typical of wild-type males during courtship. However, *cro* has not been molecularly mapped to any gene in the genome. We demonstrate that *cro* is a mutation in the gene encoding the Calmodulin-binding transcription factor (Camta) by genetic complementation tests with chromosomal deficiencies, molecular cloning of genomic fragments that flank the *cro*-mutagenic P-insertion, and phenotypic rescue of the *cro* mutant phenotype by Camta^+^-encoding cDNA as well as a BAC clone containing the gene for Camta. We further show that knockdown of the Camta-encoding gene phenocopies *cro* mutant songs when targeted to a subset of *fruitless*-positive neurons that include the mcALa and AL1 clusters in the brain. *cro-GAL4* and an anti-Camta antibody labeled a large number of brain neurons including mcALa. We conclude that the Camta-encoding gene represents the *cro* locus, which has been implicated in a species-specific difference in courtship songs between *D*. *sechellia* and *simulans*.

## Author summary

Selecting a suitable mate is a prerequisite for successful breeding in organisms. Indeed, the animals instinctively distinguish a conspecific partner from individuals of other species, yet the mechanism underlying such species-recognition remains largely unknown. In choosing a conspecific male as a mate, fruit fly females rely on a male-derived auditory signal, love song, which is generated by a series of unilateral wing vibration by the male. We study how the males produce love song that is unique to the species. We particularly focus on *croaker* (*cro*) mutants, whose males generate distorted love song. Our molecular analysis reveals that the *cro* mutation inhibits expression of the gene encoding a protein called Calmodulin-binding transcription factor (Camta) and that an introduction of the Camta-encoding DNA into the genome of *cro* mutants allows the mutant male to sing a normal song. Therefore, the Camta protein is an essential component for love song generation by males. We further show that knockdown of Camta only in tens of specific neurons in the brain is sufficient for inducing the *cro* mutant phenotype. This study paves the way for unraveling the mechanistic basis for female-male communications in conspecific mating.

## Introduction

Premating isolation between subpopulations within a species is a hallmark of incipient speciation [1]. A prerequisite to successful premating isolation is the development of a directed bias in mate choice among the members of the species. The pair of species recently separated may still be able to produce offspring between them, yet the offspring is typically infertile, representing postmating isolation [2]. *Drosophila* fruit flies offer a tractable system for studying the mechanistic basis for premating isolation due to diversified mate-choice within a population, because this genus includes the genetic model species *D*. *melanogaster*, which is highly amenable to molecular and cellular analyses for complex traits, and also because *D*. *melanogaster* has a few sibling species, with which it can produce inter-species hybrids under certain conditions, despite a strong mate choice for a conspecific partner [2–4].

Mate choice in *D*. *melanogaster* relies on multiple sensory cues [5, 6]. For example, female-derived pheromones, particularly 7,11-heptacosadiene, are important for males to discriminate conspecific females from other species [7], whereas willingness of females to mate is markedly modulated by male-derived auditory stimuli known as courtship songs [8]. Choosiness of females has been suggested to operate as one of the major drives for the assortative mating underlying premating isolation, and hence, diversification in male song patterns may result from sexual selection for favorable males [9]. These considerations point to the importance of elucidating the genetic architecture of courtship song patterning mechanisms for the understanding of behavioral evolution.

The courtship song of *D*. *melanogaster* males is generated by wing vibration [10]. The male fly laterally extends one of two wings and rapidly moves it along the anterior-posterior direction for a few seconds, followed by a series of similar vibrations made by the other wing. He alternates wings to vibrate several times in a courtship epoch directed to a target female. Phonetic recordings of courtship songs reveal two distinct song types, i.e., pulse song and sine song [11]. The pulse song is a burst of spikes with an interpulse-interval (IPI) of ~35 ms in *D*. *melanogaster* [12]. A recent analysis has captured two modes of pulse song, slow pulse and fast pulse, whose carrier frequencies are 200–250 Hz and 250–400 Hz, respectively [13]. The sine song represents sustained oscillations similar to sine waves with carrier frequencies of 120–180 Hz [11]. A classic study showed that wingless silent males succeed in copulation only at a low rate, although this poor copulation-success rate is mitigated by exposing the female to an artificial sine song [14]. Pre-exposure of females to an artificial pulse song having correct IPIs facilitates female acceptance of courting males to mate [8]. Thus, both song quality and quantity impact mating success in *D*. *melanogaster*.

The circuit for male singing has been explored in some detail. The P1 interneurons involved in courtship decision-making [15] drive, upon integrating multiple sensory inputs, P2b [16] and pIP10 [17] descending interneurons to activate the thoracic song motor pattern generator (MPG), which is composed of vPR6, vMS11, dPR1 and other interneuron groups intrinsic to the thoracic ganglia [17, 18]. When activated artificially, P1 neurons preferentially induce sine songs, whereas pIP10 neurons preferentially induce fast pulse songs [13]. In contrast to these higher brain neurons with song-triggering roles, thoracic neurons such as vPR6 and vMS11 appear to be involved in patterning of pulse songs: vPR6 artificial activation shortens IPIs; vMS11 induces unilateral wing extension without wing vibration when activated, whereas it converts monocyclic songs to polycyclic ones when inactivated [17]. The ps1 neurons are yet another component of the song motor center [19], and have been implicated in the switching between fast and slow pulse song modes [13]. Song motor outputs thus patterned are delivered to direct and indirect muscles in the thoracic segments to orchestrate wing movements for the production of a specific song type [20]. Although major neural elements for song generation have been determined as above, the detailed circuit mechanisms for the song-controlling network remain obscure.

In elucidating the neural organization and action principles of the song circuit, it is indispensable to understand the molecular constituents that play key roles in the establishment and operation of the circuit. Molecular changes in building blocks of the circuit must underlie the divergence in song characteristics among different species. Genetic screens for mutants with song pattern defects would seem to be a promising approach in unbiased searches for genes involved in song production. However, only a few mutants with abnormal courtship song structures have been isolated. *cacophony* (*cac*), a pioneering member of such mutants [12], is characterized by polycyclic pulse song and mapped to the voltage gated calcium channel gene *DMCA1A* [21]. Another mutation reported to disturb the song pattern is *dissonance* (*diss*), and males with this mutation also produce polycyclic pulse songs [22]. The *non on or off transient A* (*nonA*) gene responsible for the *diss* mutation encodes an RNA binding protein [23], which is likely required for synaptic transmission from photoreceptors as inferred from the aberrant electroretinogram (ERG) in *diss* mutants. We have isolated a P-element insertion mutant with reduced mating success, which is ascribable to defects in males but not females [24]: males of this mutant sing abnormally and thus we named it *croaker* (*cro*) [24]. In *cro* mutants, pulse song is often polycyclic, IPIs are prolonged, and the sine song amplitude within a bout is more variable [24]. However, the molecular identification of the phenotypically defined *cro* locus has been hampered by the presence of a long array of repetitive DNA sequences around the mutagenic P-element insertion. One aim of this study is to molecularly identify the gene responsible for the *cro* mutation. Upon obtaining the *cro* sequence, we can endeavor to unravel where, when and how the functional *cro* product shapes male courtship songs, using molecular probes for *cro*. This achievement of unraveling the molecular underpinning of *cro* effects on courtship songs may also shed light on the evolutionary basis for the diversification in song patterns, because *cro* is the locus that has been implicated in the species-difference of pulse song IPIs between *D*. *simulans* and *D*. *sechellia* by a Quantitative Trait Loci (QTL) analysis [25].

## Results

**Fig 1:**
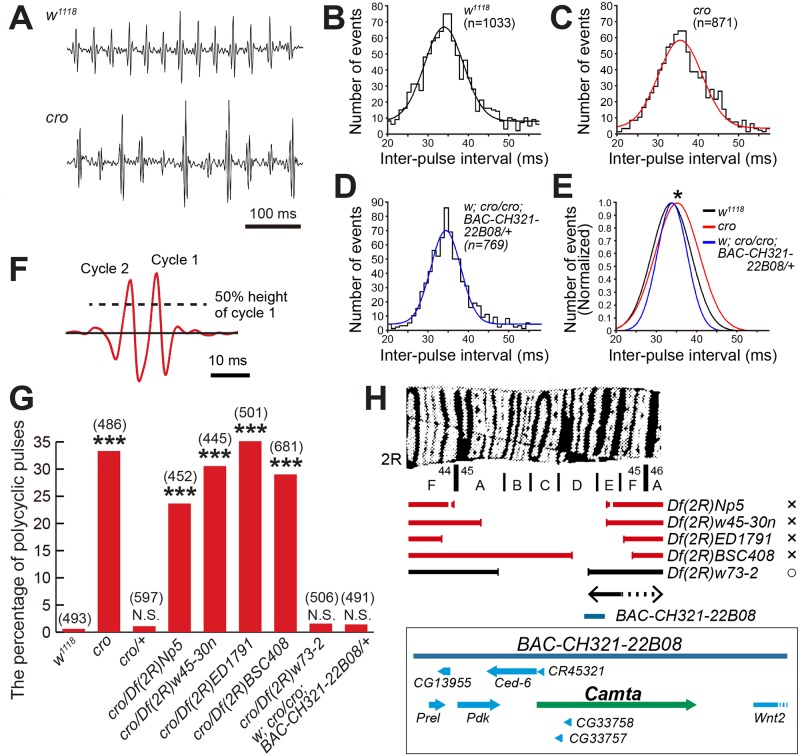
Genetic complementation tests identify the Camta-encoding region to be responsible for the *cro* mutant song phenotype. (**A**) Typical examples of pulse songs from males of the control *w*^*1118*^ (upper trace) and *cro* mutant (lower trace). (**B**-**E**) The interpulse interval (IPI) is susceptible to the *cro* mutation. (**B**-**D**) Distribution of IPIs in the control *w*^*1118*^ (**B**) and *cro* homozygote without (**C**) or with (**D**) the genomic rescue fragment *BAC-CH321-22B08*. The Gaussian fit to the IPI distribution is drawn on each histogram, and in (**E**) three Gaussian curves are superimposed on the same panel to facilitate comparisons. The statistical differences were evaluated by one-way ANOVA followed by Bonferroni’s multiple comparisons; *: P<0.05. (**F**) Criteria used to classify the recorded pulses as monocyclic or polycyclic. The peak height of the song pulse (the peak) is measured from the baseline and the 50% of this value is chosen as the slice level. When any deflections other than the primary peak exceed the slice level, the pulse is judged to be polycyclic. When the peak is the only deflection that exceeds the slice level, the pulse is judged to be monocyclic. (**G**) The incidence of polycyclic pulses compared among the genotypes indicated. The number of song pulses used to calculate the proportion of polycyclic pulses is indicated in parentheses above the bars. The proportion of polycyclic pulses is shown. The statistical significance between the control and a test group is evaluated by the Fisher’s exact probability test. ***: P<0.001, NS: not significant. (**H**) A diagrammatic representation of the genomic regions (cytology is at the top) that are deleted in deficiency chromosomes. The region covered by BAC-CH312-22B08 and the genes and their transcriptional directions are indicated by pointed bars in an expanded scale.

Because the *cro* mutant stock has been maintained for nearly 30 years without use, we first outcrossed it to a control *w*^*1118*^ strain for five generations to standardize the genetic background and examined whether the phenotype originally found in the *cro* stock can be detected in the newly outcrossed stock. We focus on the pulse song phenotype as it is highly amenable to quantitative evaluation. Indeed, the IPI prolongation reported in the original publication [24] was reproduced in this study (Fig 1A–1E). We also confirmed that the *cro* mutant males often generate polycyclic pulse song [24], which is practically absent in control male song (Fig 1A, 1F and 1G). We therefore count the incidence of polycyclic pulses as a read out of the *cro* mutant phenotype. The *cro* mutagenic P-insertion has been mapped at the cytological position 45DE by polytene *in situ* hybridization with a genomic fragment near the P-element insertion point [24]. Based on this information, we carried out genetic complement tests of the *cro* mutation with several deficiency chromosomes (*Df*s) carrying break points in this cytological region. Among the 5 *Df*s examined, *Dfw73-2* prevented polycyclic pulses from occurring, whereas *Dfs Np5*, *w45-30n*, *ED1791* and *BSC408* did not when placed in *trans* to the *cro* chromosome (Fig 1G and 1H), positioning the *cro* locus between the cytological divisions 45D8 and 45E3 (Fig 1H).

In this cytological interval, three genes with known functions have been annotated, i.e., *bruchpilot* (*brp*), *Wnt2* and *Camta*, together with additional less characterized genes as indicated in Fig 1H. To narrow our search for the genomic region responsible for the *cro* phenotype, we attempted to rescue the *cro* song defects by introducing, into the *cro* mutant line, a Bac clone containing a complete locus for *Camta* (BAC-CH321-22B08) but not functional *Wnt2* and *brp* (Fig 1H). Because BAC-CH321-22B08 was able to rescue the song defects in *cro* mutants (Fig 1D–1G), *Camta*, but not *Wnt2* and *brp*, remained a candidate for the gene responsible for the song phenotype.

**Fig 2:**
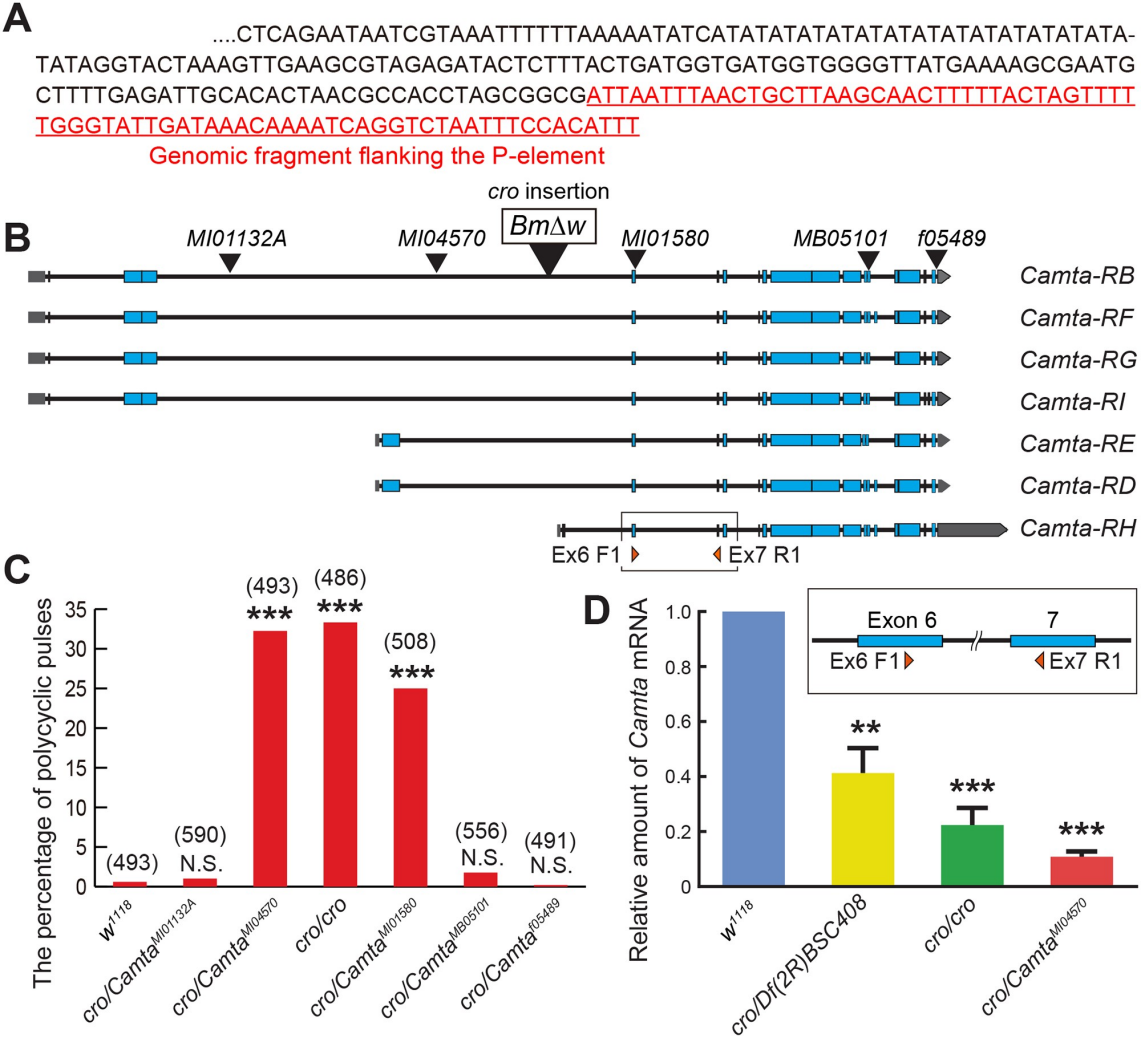
Transposon insertions into the Camta-encoding gene and their ability to induce the song phenotype when heterozygous over *cro*. (**A**) The unique sequence at the mutagenic P-element insertion in the *cro* mutant genome. The sequence cloned by plasmid rescue and inverse PCR is underlined. (**B**) The schematic drawings of the exon-intron organization for Camta splice forms and the transposon insertion points. The *BmΔw* insertion responsible for the original *cro* mutation is boxed. The positions of sequences used as primers for qPCR are indicated at the bottom and also shown in an inset between panels **B** and **D** in an expanded scale. (**C**) The incidence of polycyclic pulses compared among the genotypes indicated. The number of song pulses used to calculate the proportion of polycyclic pulses is indicated in parentheses above the bars. The proportion of polycyclic pulses is shown. The statistical significance between the control and a test group is evaluated by the Fisher’s exact probability test. ***: P<0.001, NS: not significant. (**D**) Quantitative comparisons of Camta mRNA levels among the genotypes indicated. *Camta*^*MI01580*^, which do not complement *cro*, as well as *Df(2R)BSC408* reduce Camta transcripts when placed in *trans* to *cro*. The statistical significance between the control and a test group is evaluated by the Mann-Whitney U test. ***: P<0.001, **: P<0.01, NS: not significant.

As an independent approach to the identification of the *cro* locus, we attempted to identify molecularly the P-insertion point in *cro* mutants. We took two different strategies to recover a sequence adjacent to the P-insertion site, i.e., plasmid rescue in *E*. *coli* and cloning by inverse PCR. These two approaches yielded an identical sequence that flanks the mutagenic P-element in *cro* mutants, which matched an intronic sequence in the locus encoding Camta (Fig 2A and 2B).

We note that *cac*, one of a few genes that induce polycyclic pulse song when mutated, encodes a voltage-dependent Ca^2+^ channel [21], implying that Ca^2+^ is important for the pulse song shaping, to which Camta could also contribute as a Ca^2+^-responsive transcriptional regulator. Several fly lines carrying a transposon insertion in the locus encoding Camta are publicly available (Fig 2B). We tested these five potential alleles of the *cro* locus for their ability to induce polycyclic pulse song when placed in *trans* to *cro*: *Mi{MIC}Camta*^*MI01580*^ and *Mi{MIC}Camta*^*MI04570*^ produced polycyclic pulse song, whereas *Mi{MIC}Camta*^*MI01132A*^, *PBac(WH)Camta*^*f05489*^ and *Mi{ET1}Camta*^*MB05101*^ did not (Fig 2C). We note that the three effective insertions, *cro*, *Mi{MIC}Camta*^*MI01580*^ and *Mi{MIC}Camta*^*MI04570*^, occurred at the middle of the locus, i.e., the region from exon 4 to exon 6, in contrast to the three ineffective transposon insertions, which were located more proximally (*Mi{MIC}Camta*^*MI01132A*^) or distally (*PBac(WH)Camta*^*f05489*^ and *Mi{ET1}Camta*^*MB05101*^) in the locus (Fig 2B). We inferred that the effective insertions reduce functional transcripts for Camta, thereby impairing the production of normal pulse song. Indeed, qPCR with primers specific for exon 6 and exon 7 sequences revealed a marked reduction in transcript levels for Camta in *cro*/*Mi{MIC}Camta*^*MI04570*^ as well as *cro* homozygotes and *cro/Df(2R)BSC408* (Fig 2D). None of these heteroallelic mutants caught our attention as displaying a phenotype qualitatively different from the phenotypes of the original *cro* mutants. Taking all these results together, we consider that the Camta-encoding transcription unit likely represents the *cro* locus, loss of which results in the production of polycyclic pulse song.

**Fig 3:**
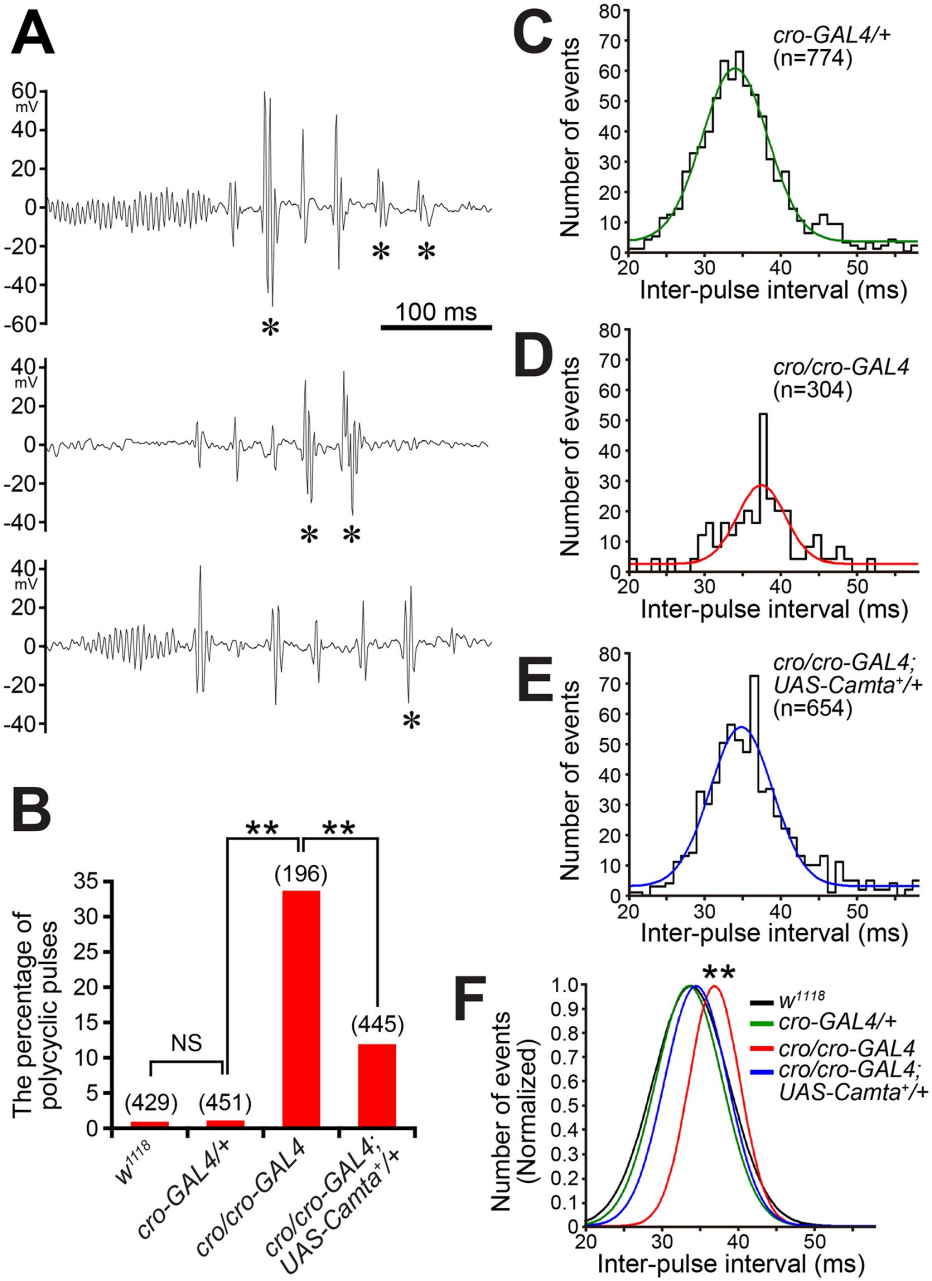
*cro/cro-GAL4* mutant song defects are rescued by Camta^+^ overexpression. (**A**) Examples of courtship song records from a *cro/cro-GAL4* mutant male. Three representative traces obtained from a single fly are shown. Pulses classified as polycyclic are marked with asterisks. (**B**) *cro* affects the incidence of polycyclic pulses. Males of *w*^*1118*^ and *cro-GAL4* rarely generate polycyclic pulses, whereas more than 30% of pulses in *cro/cro-GAL4* males are polycyclic. Overexpression of Camta^+^ rescues the mutant song phenotype. The number of pulses examined is shown in parentheses. The statistical significance of differences was evaluated by one-way ANOVA followed by Fisher’s exact probability tests; **: P<0.01. (C-F) Distribution of IPIs in the control *cro-GAL4/+* (**C**) and *cro/cro-GAL4* without (**D**) or with (**E**) the *UAS*-transgene that overexpresses Camta^+^. The Gaussian fit to the IPI distribution is drawn on each histogram, and in (**F**) three Gaussian curves and a control *w*^*1118*^ curve reproduced from Fig 1E are superimposed on the same panel to facilitate comparisons. Pulses were detected automatically as events that exceeded the slice level. In this series of experiments, the slice threshold was set at a more stringent level of 60% to avoid erroneous counts of non-song deflections due to lower signal-to-noise ratio. Even under such stringent conditions, the estimated incidence of polycyclic pulses was similar to that obtained at a slice level of 50%.

To further clarify the relationship between the Camta-encoding locus and *cro*, we generated a GAL4-expressing allele by replacing the MIMIC cassette with the GAL4-encoding sequence, i.e., *T2A-GAL4* [26], at the MI04570 insertion in the *cro* locus (S1 Fig see also Fig 2B). The *cro-GAL4* allele thus obtained was lethal, yielding no adult fly. We therefore generated *cro/cro-GAL4* heteroallelic males, which turned out to produce polycyclic song pulses (Fig 3A and 3B). The polycyclic pulses often intermingled with monocyclic pulses within a song bout; a single bout composed of exclusively monocyclic pulses or exclusively polycyclic pulses was rare (Fig 3A). The polycyclic pulses were typically of two (~65%) or three cycles (~35%), with a few exceptional pulses of more than four cycles (less than 1%). We also introduced *UAS-Camta*+ into this heteroallelic mutant, and found that the incidence of polycyclic pulses was markedly reduced (Fig 3B). This result convincingly demonstrates that deficits in the Camta-encoding gene are responsible for the song defects in *cro* mutants.

**Fig 4:**
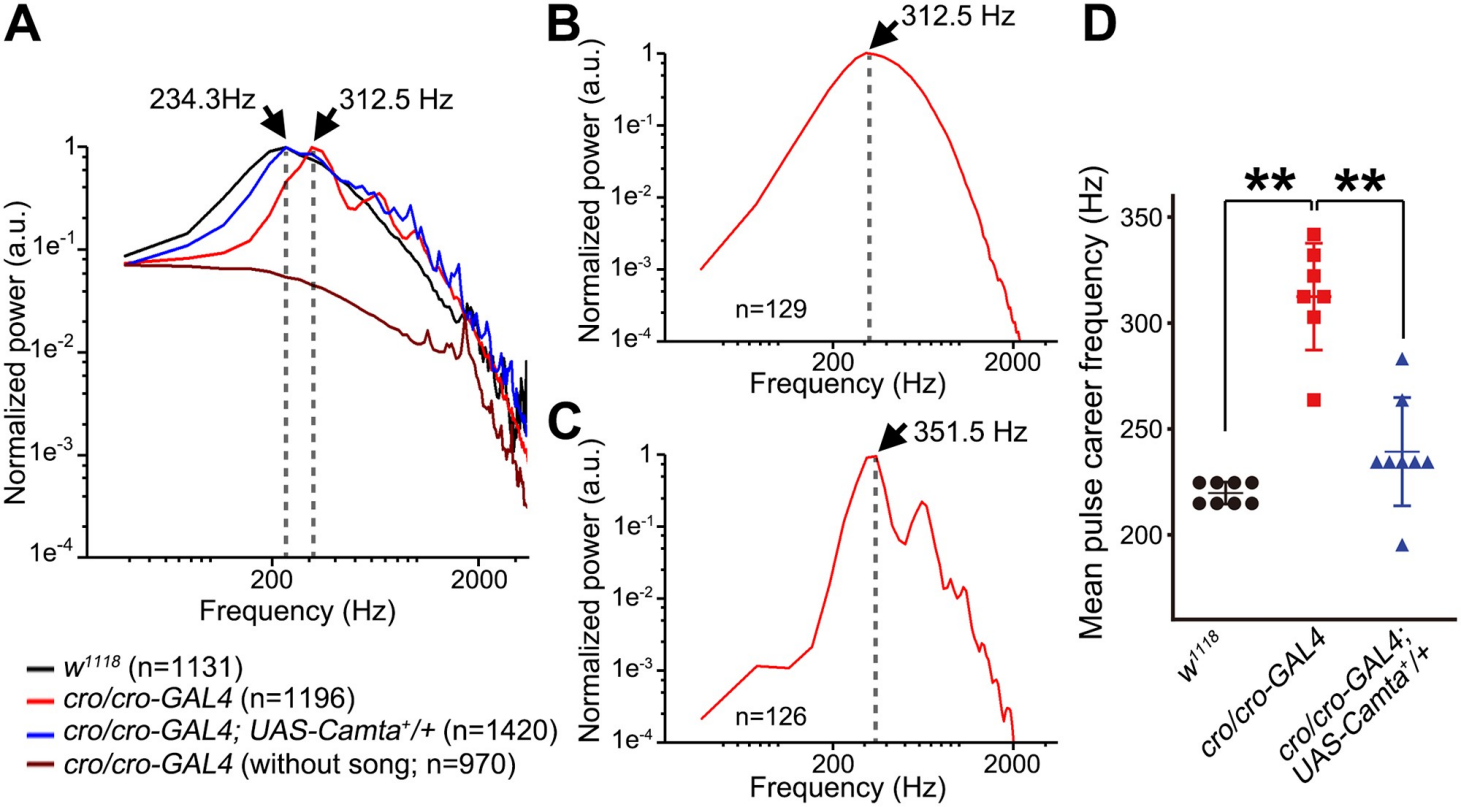
*cro* affects carrier frequency characteristics of song pulses. (**A**-**C**) Power spectra of pulse songs recorded from male flies of *w*^*1118*^ (**A**: black) and *cro/cro-GAL4* without (**A**-**C**: red) or with (**A**: blue) *UAS-Camta+*. The drawn line represents a power spectrum of traces with no song recorded from *cro/cro-GAL4*, which is indistinguishable from similar spectra of traces containing no song (i.e., noise only) obtained from other genotypes. *cro/cro-GAL4* monocyclic (**B**) and polycyclic (**C**) pulses separately subjected to fast Fourier transformation yield distinctly different power spectra. Spectra illustrated in (**B**) and (**C**) but not (**A**) have been noise-subtracted. Perpendicular broken lines indicate the maximal peak frequency. (**D**) The maximal peak frequency estimated in different flies of the indicated genotypes is compared. The means ± SEM are indicated by horizontal and vertical bars superimposed on the data points. The statistical significance of differences was evaluated by Kruskal-Wallis analysis followed by Steel-Dwass nonparametric multiple comparisons; **: P<0.01.

Because *cro/cro-GAL4* provides a useful platform for documenting and potentially rescuing mutant phenotypes, we focused on this genotype for our further analyses of song characteristics. The interpulse interval (IPI) was significantly prolonged in the *cro/cro-GAL4* song, and *Camta*^+^ overexpression restored the normal IPI in the song of this mutant (Fig 3C–3F). We then determined the carrier frequency for song pulses by fast Fourier transformation. Song pulses recorded from *w*^*1118*^ control males exhibited a conspicuous peak of spectrum density at 234.3Hz, in keeping with previous reports (Fig 4A and 4D). The power spectrum for *cro/cro-GAL4* pulses had its highest peak at 312.5 Hz, accompanied by additional, smaller peaks at even higher frequencies (Fig 4A). To evaluate the contribution of monocyclic and polycyclic pulses to the altered frequency characteristics in *cro/cro-GAL4*, we separately obtained a power spectrum for each pulse type. Monocyclic pulses resulted in a power spectrum with a peak at 312.5 Hz, followed by a monotonic decline in the power density at higher frequencies (Fig 4B). In contrast, polycyclic pulses yielded a power spectrum with the highest peak at 351.5 Hz, accompanied by multiple humps at the higher frequency range (Fig 4C). These observations indicate that the carrier frequency of mutant pulse songs is shifted to higher frequencies even in monocyclic pulses, though the magnitude of shift is smaller than in polycyclic pulses, and that multiple peaks at the higher frequency range are derived from polycyclic pulses. It is of interest to note that a power spectrum with similar multiple peaks has been reported for *diss* mutants, which also generates polycyclic pulse song [27]. All these defects in the carrier frequency of song pulses were fully rescued by Camta^+^ overexpression (Fig 4A and 4D). We conclude that the Camta-encoding gene is responsible for the *cro* song phenotypes.

**Fig 5:**
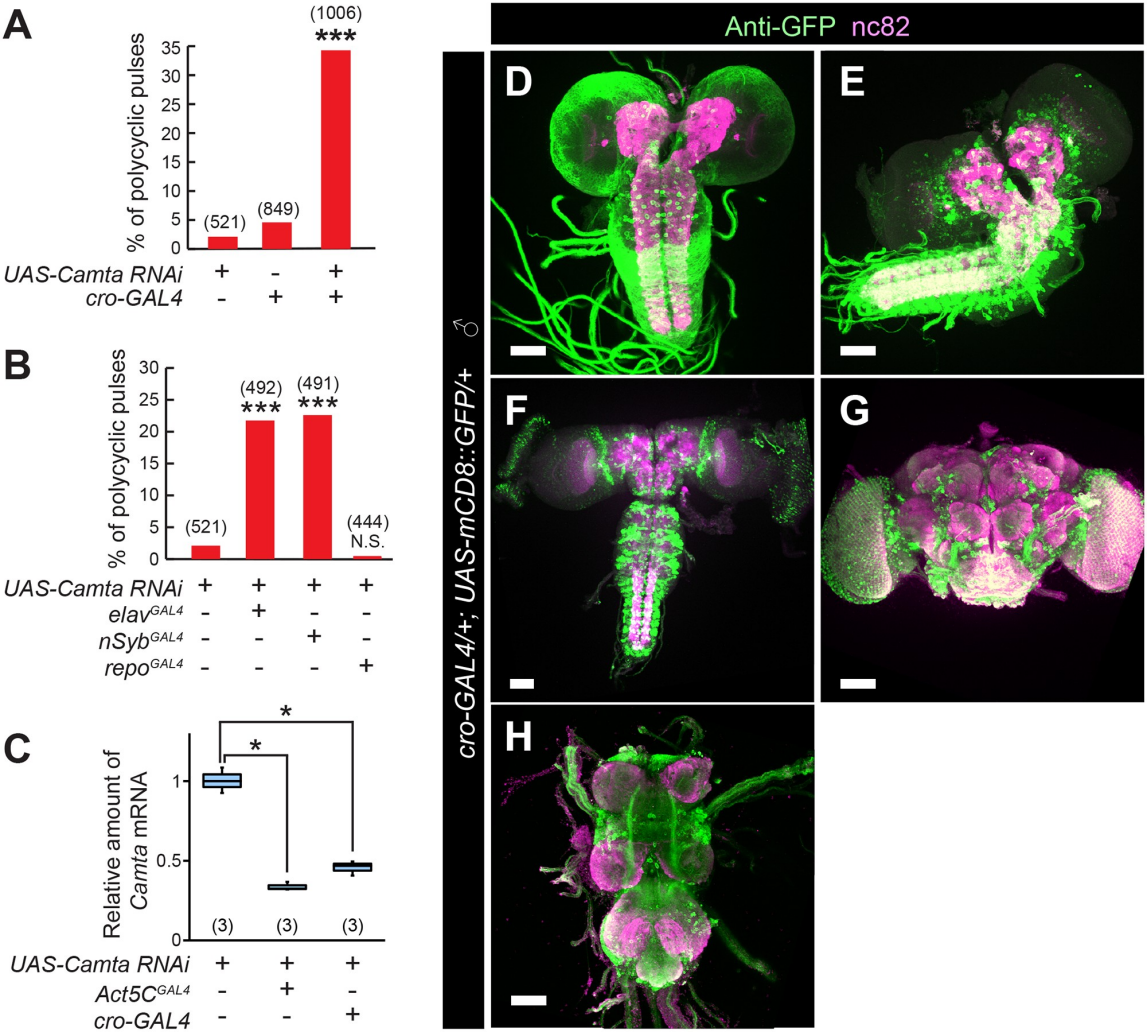
*cro* knockdown in *cro-GAL4*-positive neurons induces polycyclic pulses. (**A** and **B**) Knockdown of Camta via *cro-GAL4* (**A**), *elav*^*GAL4*^ and *nSyb*^*GAL4*^ but not *repo-GAL4* (**B**) induces polycyclic song. The proportion of polycyclic pulses was compared among the genotypes indicated. The tested flies carry transgenes marked with + at the bottom. (**B**) *fru*^*GAL4*^ but not *dsx*^*GAL4*^ is effective in inducing the song phenotype when used as a GAL4 driver. The proportion of polycyclic pulses (%) is shown in the bar chart and the number of pulses analyzed is indicated in parentheses. Statistical significance was evaluated by the Fisher’s exact probability test. ***: P< 0.001; NS: not significant. (**C**) qPCR reveals a reduction of Camta transcripts by RNAi-mediated knockdown as driven by *Act5C-GAL4* or *cro-GAL4*. The flies examined carried the transgenes indicated by the plus mark below the box plot graph. The number of replicates is shown in parentheses. The box plot shows the median and 10^th^, 25^th^, 75^th^, and 90^th^ percentiles. The statistical significance of differences was evaluated by Kruskal-Wallis analysis followed by Steel’s nonparametric multiple comparisons: *: P<0.05. (**D**-**H**) *cro-GAL4* expression is widespread in the central nervous system at the third instar larva (**D**), wandering stage larva (**E**), pupa (**F**), and adult stages (brain: **G**; VNC: **H**). Scale bars: 50 μm (**D**-**H**).

**Fig 6:**
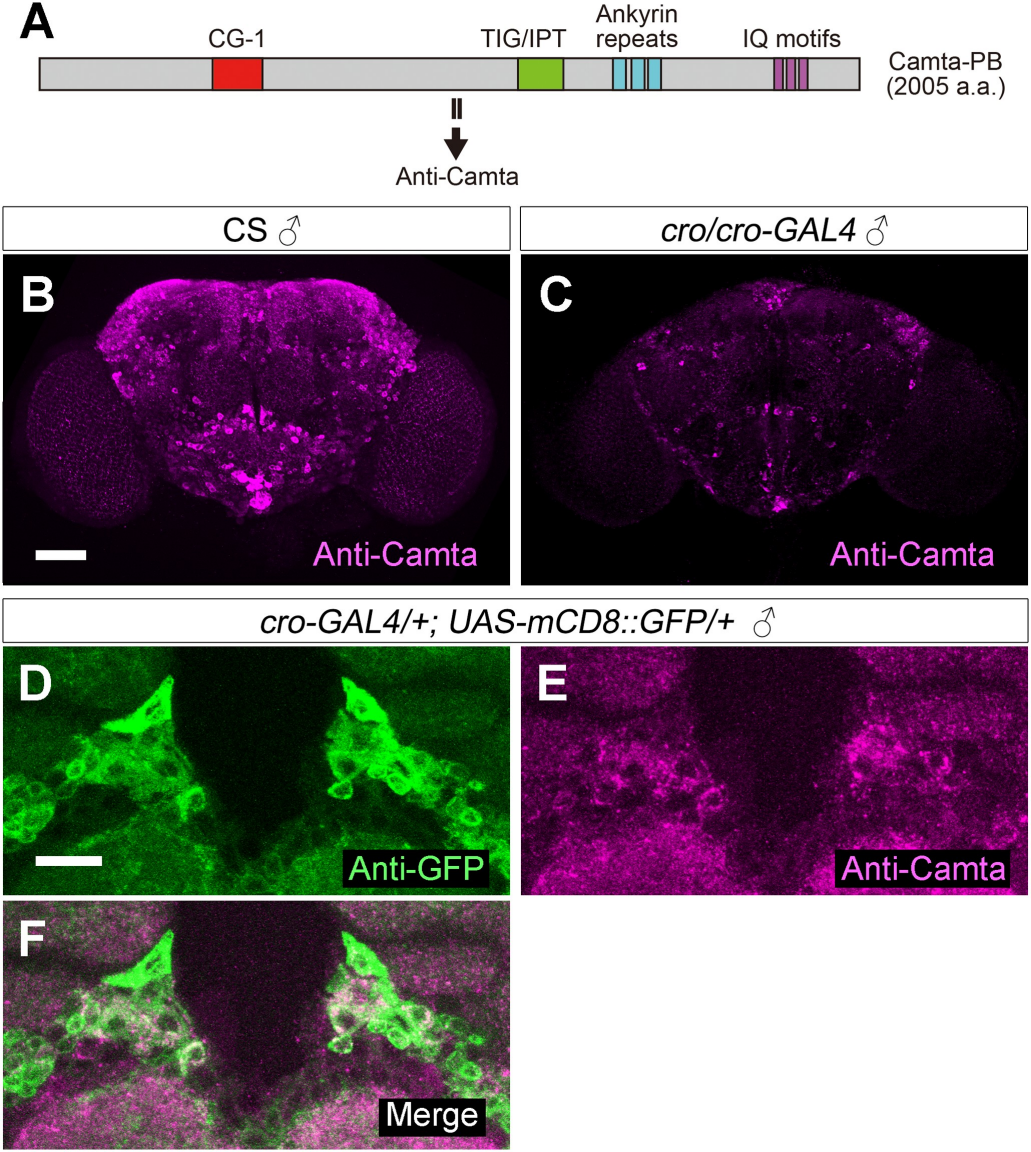
Expression of *cro-GAL4* recapitulates endogenous Camta expression. (**A**) The antibody recognizes a unique sequence in Camta, whose domain structure is schematically illustrated. (**B** and **C**) The entire brain stained positive for Camta in wild-type (**B**) and *cro/cro-GAL4* (**C**) males. (**D**-**F**) High magnitude images of the periesophageal region highlight cell bodies doubly positive for GFP (**D** and **F**: *cro-GAL4*, green) and Camta (**E** and **F**, magenta). Scale bars: 50 μm (**B**; also applicable to **C**) or 10 μm (**D**; also applicable to **E** and **F**).

As expected, *cro-GAL4*-driven Camta knockdown reduced Camta mRNA expression and yielded male flies that generated polycyclic pulse song during courtship (Fig 5A and 5C). To determine the tissue in which the Camta-encoding *cro* gene functions for the shaping of pulse song, we carried out knockdown of the Camta-encoding gene as targeted to neurons, a cell type where *cro* likely functions in male flies to generate normal pulse song. Two neural drivers, *elav*^*GAL4*^ and *nSyb*^*GAL4*^, both induced polycyclic song, whereas the glial driver *repo*^*GAL4*^ did not (Fig 5B), supporting the hypothesis that the gene for Camta is responsible for the *cro* song defects and is required in neurons for normal song generation. In accord with this hypothesis, *cro-GAL4* was widely expressed in the nervous system at the larval, pupal and adult stages (Fig 5D–5H; S2A–S2E Fig). A newly generated antibody against Camta (Fig 6A) labeled a large number of cells in the brain, the staining intensity of which was reduced in *cro/cro-GAL4* mutants (Fig 6B vs. 6C). The majority of brain cells positive for the anti-Camta antibody expressed *cro-GAL4*, indicating that *cro-GAL4* faithfully recapitulates endogenous Camta expression (Fig 6D–6F).

**Fig 7:**
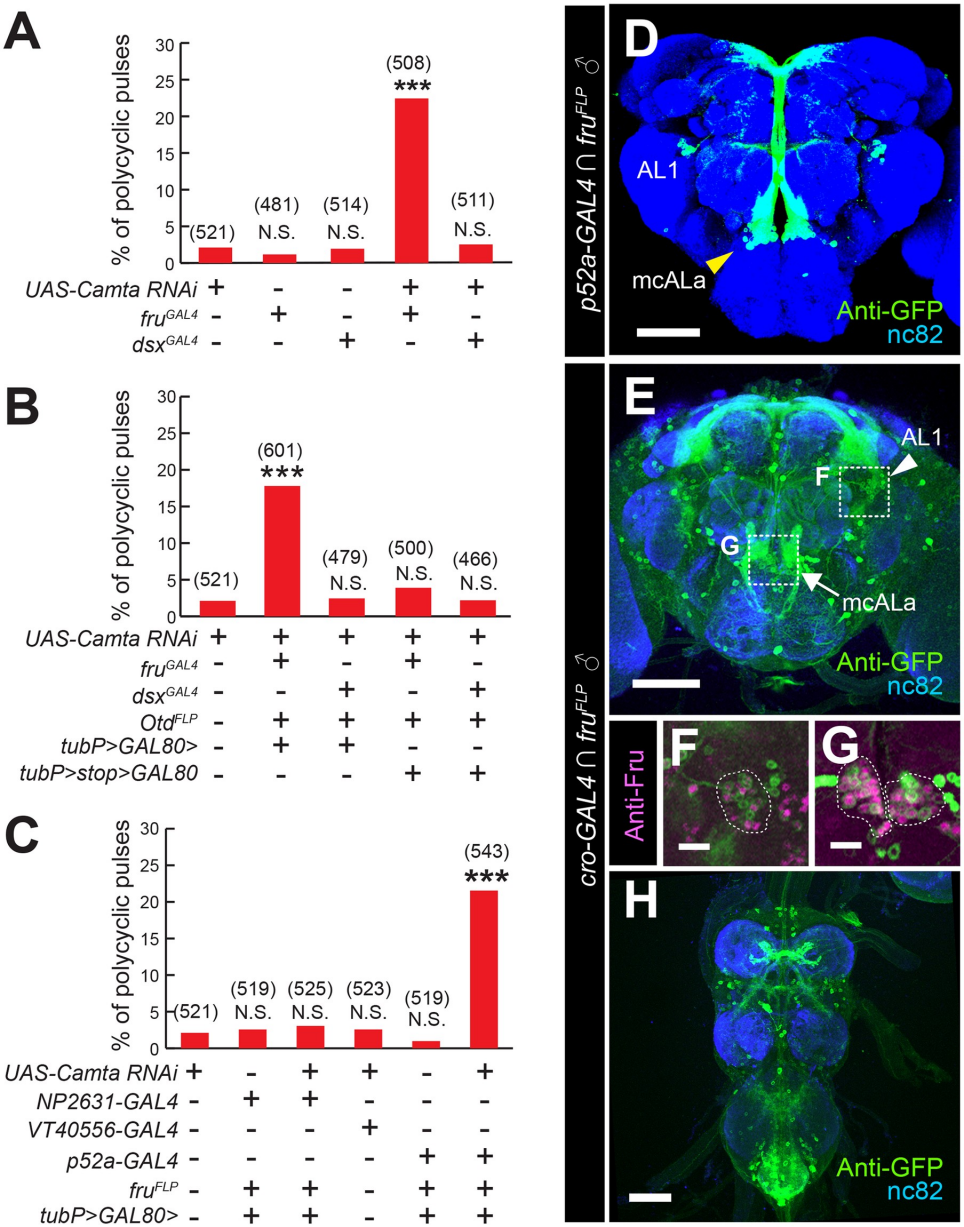
*cro* functions in the *fru*^*GAL4*^-positive but not *dsx*^*GAL4*^-positive population of neurons for normal song production. (**A**) *fru*^*GAL4*^ but not *dsx*^*GAL4*^ is an effective *GAL4* driver for *cro* knockdown in inducing the song phenotype. (**B**) When *Otd*^*FLP*^ was used to restrict the effects of *fru*^*GAL4*^ on neurons in the brain, polycyclic pulse generation was not inhibited, suggesting that brain *fru*-expressing neurons are involved in the observed song phenotype. (**C**) The combination of *p52a-GAL4* and *fru*^*FLP*^ allows mcALa-specific expression of *Camta* RNAi, resulting in the polycyclic pulse generation. P1-specific *NP2631-GAL4* and pIP10-specific *VT40556-GAL4* are without effect on the pulse song shape when used as drivers for *Camta* RNAi. (**D**) Brain neurons visualized by *UAS>stop>mCD8*::*GFP* as driven by *p52a-GAL4* in the presence of *fru*^*FLP*^. mcALa neurons and a few additional *fru*-positive clusters including AL1 are stained. (**E-H**) *cro-GAL4* expression in the nervous system includes *fru*-positive mcALa and AL1 neurons. *cro-CAL4* and *fru*^*FLP*^ intersection visualized mcALa and AL1 neurons. mcALa (arrows) and AL1 (arrowheads) are shown in the entire brain (**E**) or in enlarged views (**F** and **G**). AL1 in the right hemisphere (**F**) and bilateral mcALa (**G**) are delineated with white broken lines. The intersection also stained a large number of *fru-GAL4*-positive neurons in the VNC (**H**). The antibodies used for staining are indicated above each panel. Scale bars: 50 μm (**D**, **E** and **H**) or 10 μm (**F** and **G**).

The fact that a neuronally targeted knockdown of the gene encoding Camta phenocopied *cro* mutants suggests an essential role of Camta in neurons for song patterning. To determine which subset of CNS cells requires functional *cro* in shaping pulse song, we performed *cro* knockdown in *fru*^*GAL4*^- or *dsx*^*GAL4*^-positive cells, because the core portion of the male courtship circuit is composed of *fru*- and/or *dsx*-expressing neurons [18, 28]. Notably, *fru*^*GAL4*^-driven *Camta* RNAi expression, but not *dsx*^*GAL4*^-driven *Camta* RNAi expression, induced polycyclic pulse song, indicating that *fru*^*GAL4*^-positive neurons contribute, at least in part, to *cro*-dependent shaping of pulse song patterns (Fig 7A). It has been postulated that courtship decisions are made in the brain [15], whereas the motor pattern generator for courtship behavior resides in the ventral nerve cord (VNC) [17]. To clarify which of these centers (brain vs. VNC) requires *cro* for the production of monocyclic pulse song, we restricted *Camta RNAi* expression to *fru*^*GAL4*^-positive neurons in the brain by the use of *Otd*^*FLP*^ [29], which releases GAL4 from GAL80-mediated repression by flipping the *GAL80* sequence (*>GAL80>*) out from *tub>GAL80>* only in the head. *Otd*^*FLP*^ effectively restricted *fru*^*GAL4*^-driven *UAS-mCD8*::*GFP* expression in the brain (S3 Fig). We found a significant increase in the incidence of polycyclic pulse song in male flies in which *cro* knockdown was restricted to *fru*^*GAL4*^-positive neurons in the brain (Fig 7B; S2F–S2K Fig), supporting the notion that *cro*-positive *fru*-expressing brain neurons play an important role for pulse song shaping. However, polycyclic pulses were less frequent when *cro* knockdown was restricted to the brain *fru*-expressing neurons than when *cro* knockdown was achieved in wider domains of tissues as driven by, for example, *cro-GAL4*, *elav*^*GAL4*^ or *nSyb*^*GAL4*^(Fig 5B cf. Fig 7B). *fru*-positive neurons in the VNC, *fru*-negative cells in the nervous system and even non-neuronal cells might also be involved, to some extent, in the song defects.

Taking into account the observation that the head-restricted *cro* knockdown in *fru*^*GAL4*^-expressing neurons induced polycyclic pulse song, we next tested whether *cro* knockdown in a few *fru*-expressing brain neurons known to have a pivotal role in courtship song generation can induce polycyclic pulse song. We first examined pIP10 neurons which represent laterally paired descending interneurons with a command fiber-like function to initiate male courtship upon artificial activation. These interneurons have been reported to trigger pulse song generation when forcibly activated [13]. Notably, *cro* knockdown specifically targeted to pIP10 neurons by *VT40556-GAL4* did not induce polycyclic pulse song during male courtship toward a female (Fig 7C). Next, we examined the effect of *cro* knockdown in mcALa neurons, which have been implicated in the ordering of courtship elementary actions [30]. Interestingly, the mcALa-specific intersectional combination *p52a-GAL4* and *fru*^*FLP*^ led to the generation of polycyclic pulse song (Fig 7C and 7D), suggesting a new role for mcALa, i.e., shaping pulse song. We note, however, that this intersectional combination also induced *Camta* RNAi expression in a few additional *fru*-positive cells, including AL1. We cannot exclude the possibility that these cells, rather than mcALa, are responsible for the polycyclic pulse song generation. In contrast, P1-targetted *cro* knockdown by the intersection of *NP2631-GAL4* and *fru*^*FLP*^ did not induce polycyclic pulse song during male courtship (Fig 7C). Importantly, we detected *cro-GAL4* expression in mcALa and AL1 by the intersection of *cro-GAL4* and *fru*^*FLP*^ (Fig 7E–7H). The anti-Camta antibody strongly labeled cell bodies around the suboesophageal ganglion (SOG), which appeared to include those of mcALa (Fig 6D–6F). We were unable to distinguish AL1 somata from surrounding cell bodies labeled by the anti-Camta antibody. We conclude that *cro* is required in some *fru*-expressing neurons, including mcALa, and that the polycyclic nature of pulse song is a manifestation of malfunction of these neurons that constitute the core brain circuit for male courtship behavior.

## Discussion

Camta represents a conserved family of proteins known to exist in a wide range of phyla from plants to mammals [31, 32]. The Camta proteins are characterized by the DNA-binding CG-1 domain at the N-terminus and the IQ domain for interacting with Calmodulin at the C-terminus [33, 34]. Camta has been shown to bind to a DNA motif known as the CGCG-box and transcriptionally activates a nearby gene [33, 34]. In *Drosophila*, Camta has been shown to promote expression of *dFbxl4*, which encodes a transcription factor required for rapid deactivation of rhodopsin [35, 36].

In this study, we presented evidence that the polycyclic distortion of pulse song in *cro* mutant males originates from defects in the *fru*-labeled circuit underlying male courtship behavior. Of note, loss of *cro* from mcALa neurons was sufficient to induce a discernible change in pulse song. mcALa neurons originate from the SOG and send midline ascending axons to the dorsal protocerebrum, contributing part of the so-called arch [37]. An intriguing possibility is that synaptic connections formed by these neurons with other cells within the courtship circuit are disturbed by loss of *cro*. Another interesting possibility is that mcALa neurons need to acquire some physiological properties essential for the song shaping function.

Whereas mcALa neurons are confined in the brain-SOG complex (Fig 7D), song motor outputs are likely produced in the MPG localized in the thoracic ganglia [17]. It is envisaged that neurons composing the MPG require, in order to shape pulse song, mcALa-derived tuning signals, which are probably relayed as yet unidentified descending neurons to the MPG from the brain-SOG complex [38, 39]. Ding et al. [40] recently showed that artificial activation of pIP10 descending neurons can produce a high frequency clack song, similar to that generated by *D*. *yakuba* males, in *D*. *melanogaster*, which usually do not produce this type of song. They postulated that fine adjustments of the existing descending system, e.g., strengthening synaptic connections with an otherwise silent MPG, could result in a new behavioral trait (e.g., song) in a derived species through evolution [40]. By analogy, one might envisage that *cro* is required in order to adjust the strength of connectivity between mcALa neurons and a descending system that drives a pulse song MPG. In light of the suggested mcALa function, i.e., aligning courtship elements in sequence, this neuronal group is anticipated to have access to multiple MPGs, making it an ideal site where quantitative changes in a courtship action are produced. In regard to the contracted courtship behavior reported to occur upon *fru* knockdown in mcAL (including mcALa) neurons [30], mcALa-targeted *Camta* knockdown or *cro* mutations did not impair the sequential execution of courtship behavior. It might be that, in shaping pulse song, *cro* functions independently of *fru* gene products, which are nonetheless required for ordering courtship actions in correct sequence. The precise characterization of deficits in neural structure and function caused by *cro* loss is an urgent issue to be addressed.

The song structure and organization are species-specific and such divergence in courtship songs is indeed key for reproductive isolation among species [41, 42]. One of the most striking cases of song-dependent sexual isolation is found between *D*. *montana* and *D*. *lummei*, two closely related species that are sympatric in some habitats, which do not mate with each other in nature [41]. However, wing-removed and thus silent males of *D*. *lummei* were able to copulate with *D*. *montana* females, provided that pulse song with the *montana*-specific IPI was played back in mating assays [41]. In contrast, pulse song with the *limmei*-specific IPI had no “rescuing” effect [41]. QTL analysis of the IPI difference between two *melanogaster* relatives, *D*. *simulans* and *D*. *sechellia*, suggested *cro* in addition to *maleless* and *fru* as candidate loci for the diversification in this trait [25]. Molecular identification of *cro* by the present work will pave the way to the determination of potential changes in the song shaping function of this gene in evolution, thereby unveiling the mechanistic basis underlying precopulatory isolation in incipient speciation.

## Material and methods

### *Drosophila* stocks

Flies were reared on a cornmeal-yeast medium under a 12:12 light:dark cycle at 25 °C, except when specifically stated. The following strains were established or kept as lab stocks in our laboratory: the wild-type *Canton-S*, *w*^*1118*^, *cro*, *elav*^*C155*^ *(elav*^*GAL4*^*)*, *nSyb*^*GAL4*^, *repo*^*GAL4*^, *Act5C*^*GAL4*^ and *UAS-mCD8*::*GFP*. The following strains were obtained from the Bloomington stock center: *Df(2R)Np5* (#3591), *Df(2R)w45-30n* (#4966), *Df(2R)w73-2* (#6247), *Df(2R)BSC408* (#24912), *Camta*^*MI01580*^ (#32766), *Camta*^*MI04570*^ (#37957), *Camta*^*f05489*^ (#18874), *Camta*^*MI01132A*^ (#61689), *Camta*^*MB05101*^ (#24606), *UAS-Camta-RNAi* (#35683, #40849), *tubP>GAL80>* (#38879, #38880) and *tubP>stop>GAL80* (#38878). *UAS-Camta*^+^ (#F000702) strain was obtained from the Zurich ORFeome Project (FlyORF). *Df(2R)ED1791* (#150124) and *NP2631* (#104266) strains were obtained from Kyoto Stock Center. *BAC-CH321-22B08* strain was obtained from BACPAC Resources Center (BPRC). *VT40556* (#201129) strain was obtained from the Vienna *Drosophila* Resource Center. *Otd-FLP* was the kind gift from David J. Anderson (California Institute of Technology). *fru*^*GAL4*^, *fru*^*FLP*^, *UAS>stop>mCD8*::*GFP*, and *p52a-GAL4* strains were the kind gifts from Barry Dickson (Janelia Farm). *dsx*^*GAL4*^ was the kind gift from Stephen F. Goodwin (University of Oxford).

### Behavioral assays

The virgin males and females were collected at eclosion. Males were placed singly in food vials, while 10 females were placed together in single food vials. They were kept at 25 °C until being subjected to song recording, which was carried out on males aged 5–7 days after eclosion. The male was placed in a small chamber (0.8 cm in diameter, 0.3 cm in height) with a wild-type virgin female (3–5 days after eclosion). Courtship songs were recorded *via* a microphone (NR-23160; Knowles) placed beneath the chamber as described previously [43]. The sound signal from the microphone was amplified by a differential amplifier (DP-304; Warner Instruments) and recorded by an AD converter (ML826 PowerLab 2/26; AD Instruments). The maximal height of a song pulse was measured between the largest positive or negative peak and the baseline, which was defined as the mean output voltage of a 3-sec period that immediately preceded the rising phase of the relevant deflection (in contrast to the conventional definition of the amplitude of a pulse, which refers to the distance between the largest positive and negative peaks). To determine the number of cycles within a pulse, we set the slice level at 50% of the maximal height of each pulse unless otherwise specified, and if any deflections other than the primary (largest) peak exceeded the slice level, those deflections were counted as auxiliary peaks. When a song pulse had no additional peak that exceeded the slice level (other than the primary peak), it was classified as monocyclic. When a song pulse had one or more auxiliary peak(s), it was considered to be polycyclic. Song pulses in arbitrarily chosen segments of song records from 10 male flies of a given genotype were used to calculate the proportion of the polycyclic events. Our method to determine song pulse cycles was adapted from Rendahl et al. [44], where the slice level was set at 25% rather than 50%. The change from 25% to 50% will reduce false-positive counts due to the superposition of noise on a song pulse. Songs were typically recorded for 10 min from a given fly. To obtain IPI histograms and power spectra for song pulses, song records obtained as above were fed into a Digidata 1440A digitizer (Molecular Devices), followed by the analysis of relevant parameters using Clampfit 10.6 software (Molecular Devices). For fast Fourier transformation, digitized song records were treated at the sampling rate of 10,000 Hz, after filtering low frequency components at ~ 1 Hz and high frequency components at ~500 Hz.

### Molecular cloning of the *cro* gene by plasmid rescue

The genomic DNA fragment near the *cro* locus was cloned by plasmid rescue [45] using the plasmid sequence contained in the *BmΔw cro* mutagenic P-element. Genomic DNA was purified from *cro* homozygous flies using DNeasy Blood & Tissue Kit (Qiagen, 69504). 1 μg genomic DNA was digested overnight with *Eco*RI (New England Biolabs, R3101) and circularized with the T4 DNA ligase at room temperature for 1 hour, then transformed into JM109 *E*. *coli* competent cells (Nippongene, 310–06246). The genomic fragment surrounding the site of *BmΔw* insertion was sequenced with a primer BmΔw-F1 (5’-ATTCAGTGCACGTTTG-3’) using ABI 3500 Genetic Analyzer (Applied Biosystems).

### Inverse PCR

Inverse PCR was performed as previously described [46]. Genomic DNA from *cro* homozygous flies was cut with *Bam*HI (New England Biolabs, R0136), diluted, and circularized with the T4 DNA ligase. The BmDw-F1 forward (5’-ATTCAGTGCACGTTTG-3’) and BmDw-R4-3 reverse (5’-TGTCCGTGGGGTTTGA-3’) primers were used to amplify the flanking DNA to the 3’ end of the *BmΔw* P-element. The PCR product thus obtained was directly sequenced with ABI3500 Genetic Analyzer (Applied Biosystems).

### qPCR

qPCR was performed using a LightCycler 1.0 system (Roche). Total RNA was extracted from the head of 10 male adults of the indicated genotype using an RNeasy Mini Kit (Qiagen, 74104). To quantify Camta transcript expression levels, RNA was converted into cDNA using a ReverTra Ace qPCR RT kit (TOYOBO, FSQ-101). cDNA was mixed with SYBR Premix Ex Taq II (TAKARA, RR820S) and 5 pmol of both forward (5’-AGCCGACAGTTTTCCATCAC-3’) and reverse (5’-CACTCGCCATGCTTATCAAA-3’) primers. *RpL32* (*rp49*) was amplified as an internal control using the primer pair 5’-AGATCGTGAAGAAGCGCACCAAG-3’ (forward) and 5’-CACCAGGAACTTCTTGAATCCGG-3’ (reverse). qPCR was conducted at 95 °C for 30 sec (initial denaturation), followed by 40 cycles of denaturation at 95 °C for 5 sec, annealing at 55 °C for 30 sec and elongation at 72 °C for 30 sec. Data processing was performed using LightCycler Software Ver. 3.5 (Roche).

### Generation of the *cro-GAL4* transgenic fly line

To obtain the *cro-GAL4* driver, we employed recombinase-mediated cassette exchange (RMCE) [47] at an MiMIC insertion within the *cro* locus. The *GAL4*-containing plasmid vector *pBS-KS-attP2-SA(1)-T2A-GAL4-Hsp70* was injected together with ΦC31 plasmid DNA into the embryos of flies bearing the *Mi{MIC}04570* insertion in the *cro* locus. The recombinant transformant was signified by loss of the *yellow*^+^ selection marker, and the orientation of the *GAL4* insertion was determined by genomic PCR using the PCR primers Camta F1 (5’-GCTTCGGAAATCATGCTGTA-3’) and GAL4 R103 (5’-CACTTGGGCTTCTCCTTGCT-3’).

### Generation of anti-Camta antibodies

A rabbit polyclonal anti-Camta antibody was raised against a mixture of two 14-mer peptides, TVNNQATEAPNRSQ and YSASSDNSSQISDE, which were both encoded by exon 13 of the Camta-encoding gene (residues 1046–1059 and 1077–1090 of Camta (GenBank accession number ACL83079)), and was affinity purified.

### Immunohistochemistry

The central nervous system (CNS) was dissected from flies in PBS, and fixed in 4% paraformaldehyde dissolved in PBS for 60 min. Immunostaining was carried out as described previously [16], using the following antibodies at the indicated dilutions: rabbit anti-GFP (Sigma, A6455; 1:500), mouse Anti-Brp (nc82) (DSHB, 1:10), guinea pig anti-FruMale [48] (1:500) and rabbit anti-Camta (this study, 1:200). The secondary antibodies used were as follows: Alexa488-conjugated goat anti-rabbit IgG (Thermo Fisher, A-11034) at 1:500, Alexa546-conjugated goat anti-mouse, rabbit or guinea pig IgG (Thermo Fisher, A-11003, A-11035 or A-11074) at 1:500, and Alexa647-conjugated goat anti-mouse IgG (Thermo Fisher, A32723) at 1:500. Stacks of optical sections were obtained with a Zeiss LSM 510 META confocal microscope and were processed with Image J software.

## Supporting information

S1 Fig Generation of *cro-GAL4*. Top: the exon-intron organization and the insertion points of the MIMIC (*MI04570*) and *cro* P-element (*BmΔw*) in the *cro* locus. Middle: a schematic illustrating the structure of MIMIC composed of *Minos* inverted repeats (MiL and MiR), splice acceptor sites (SA), inverted ΦC31 *attP* sites (attP), stop-start sequence (3xStop), fluorescent marker *EGFP*, *polyA* and visible marker *yellow*^+^. The *pBS-KS-attP2-SA(1)-T2A-GAL4-Hsp70* plasmid vector used for replacing MIMIC with *T2A-GAL4* via recombination at attP and attB. SD: splice donner site.
(TIF)

S2 Fig *cro-GAL4* expression in the central nervous system. (**A**-**E**) *cro-GAL4* expression in the larval, pupal and adult stages. Panels **A** to **E** are duplicates of panels **D** to **H** of Fig 5, respectively, except that only GFP signals are shown here with no superposition of nc82 signals. (**F**-**K**) Visualization with GFP (green) of cells specified by the *cro-GAL4* and *fru*^*FLP*^ intersection in the brain (**F**-**H**) and VNC (**I**-**K**). An anti-Fru antibody (magenta) labels many of these cells.
(TIF)

S3 Fig *Otd*^*FLP*^ effectively restricts *fru-GAL4* expression to the brain. *fru-GAL4* is expressed in the brain regardless of whether the *Otd*^*FLP*^ + *tubP>GAL80>* cassette is present (**A**) or absent (**B**), whereas its expression in the VNC is only detected in the absence of the cassette in flies that carry *UAS-mCD8*::*GFP* and *fru-GAL4*. The tissues were stained with an anti-GFP antibody for *fru-GAL4* (green) and counterstained with nc82. Scale bar: 50 μm.
(TIF)

S1 Table Genotypes of flies used in this study. (DOCX)

S2 Table Numerical data underlying graphs. (XLSX)
