# Supplementary figures and images for: Calmodulin-binding transcription factor shapes the male courtship song in Drosophila
Source: PLoS Genet. 2019 Jul 25;15(7):e1008309. doi: 10.1371/journal.pgen.1008309 (PMC6690551; doi:10.1371/journal.pgen.1008309)

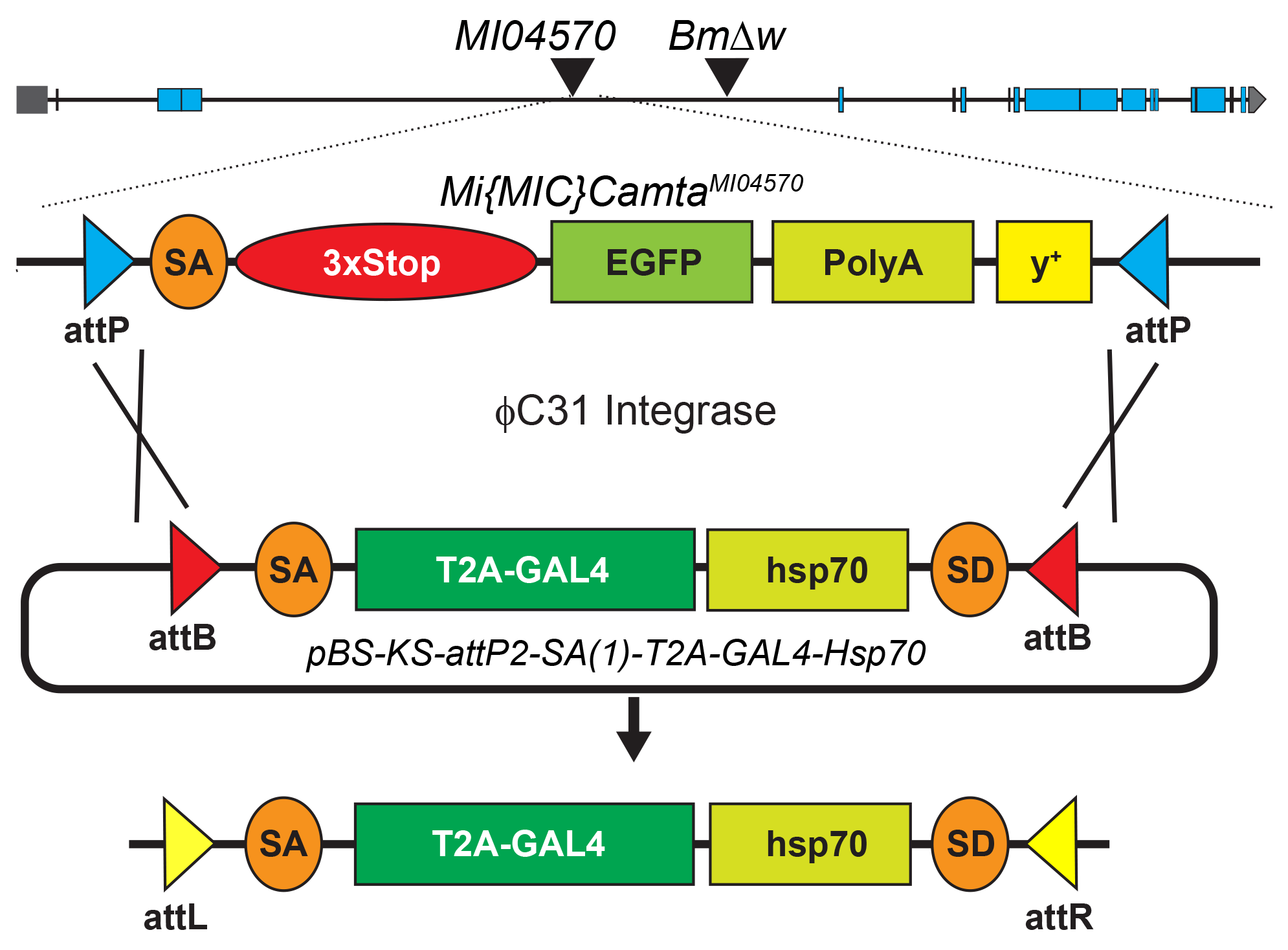

Supplement: S1 Fig — Top: the exon-intron organization and the insertion points of the MIMIC (MI04570) and cro P-element (BmΔw) in the cro locus. Middle: a schematic illustrating the structure of MIMIC composed of Minos inverted repeats (MiL and MiR), splice acceptor sites (SA), inverted ΦC31 attP sites (attP), stop-start sequence (3xStop), fluorescent marker EGFP, polyA and visible marker yellow+. The pBS-KS-attP2-SA(1)-T2A-GAL4-Hsp70 plasmid vector used for replacing MIMIC with T2A-GAL4 via recombination at attP and attB. SD: splice donner site. (TIF) [file pgen.1008309.s001.tif]

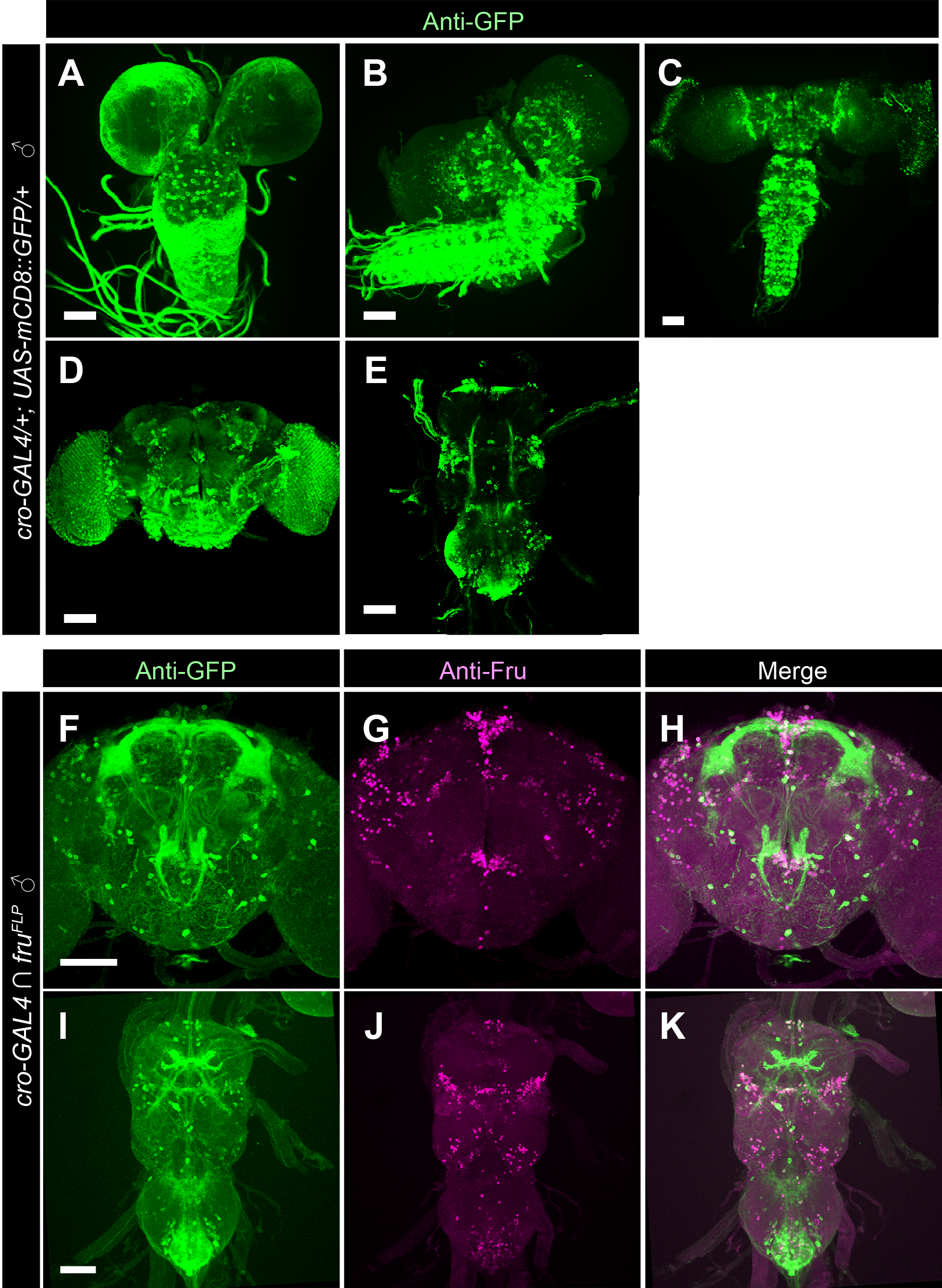

Supplement: S2 Fig — (A-E) cro-GAL4 expression in the larval, pupal and adult stages. Panels A to E are duplicates of panels D to H of Fig 5, respectively, except that only GFP signals are shown here with no superposition of nc82 signals. (F-K) Visualization with GFP (green) of cells specified by the cro-GAL4 and fruFLP intersection in the brain (F-H) and VNC (I-K). An anti-Fru antibody (magenta) labels many of these cells. (TIF) [file pgen.1008309.s002.tif]

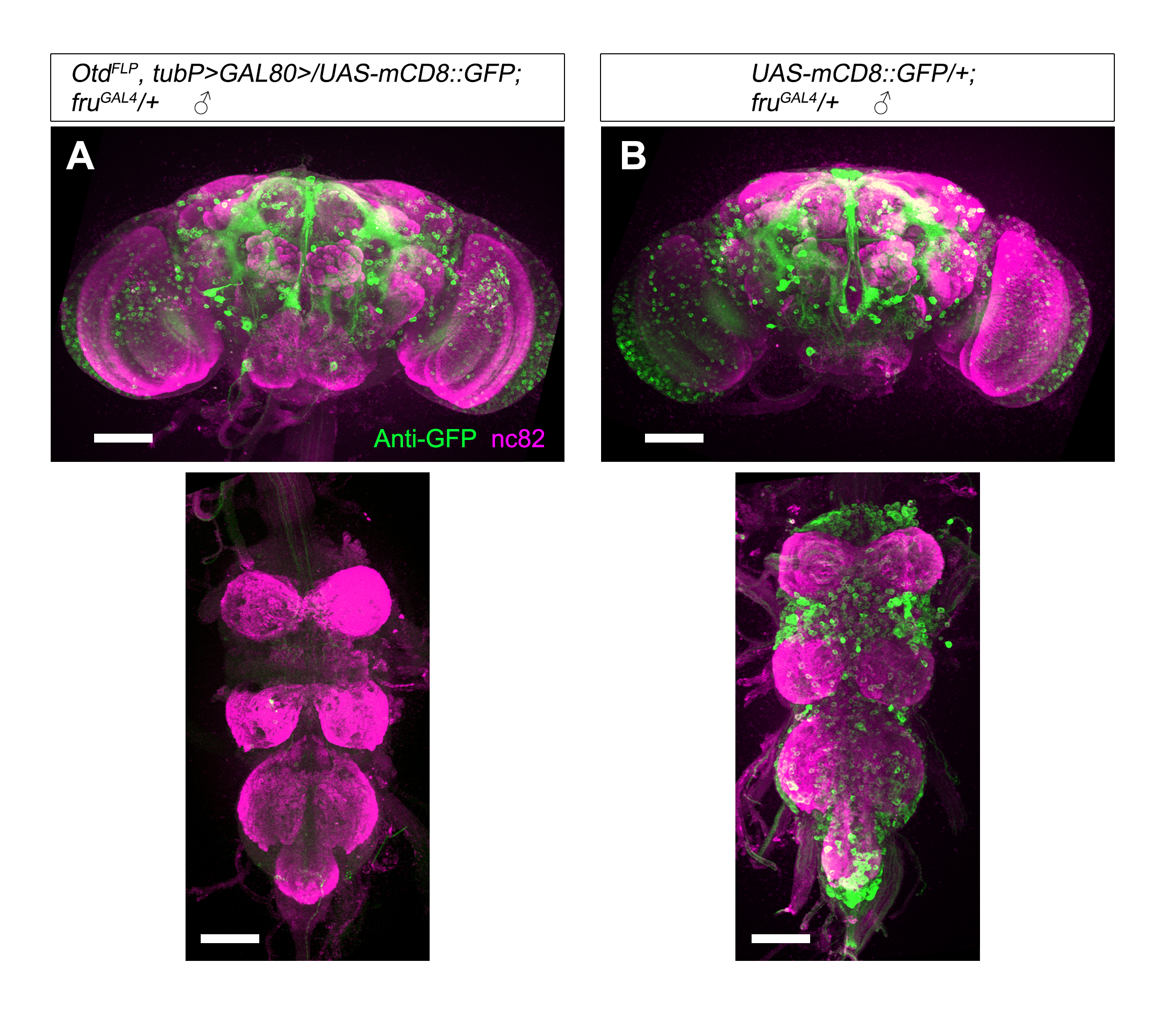

Supplement: S3 Fig — fru-GAL4 is expressed in the brain regardless of whether the OtdFLP + tubP>GAL80> cassette is present (A) or absent (B), whereas its expression in the VNC is only detected in the absence of the cassette in flies that carry UAS-mCD8::GFP and fru-GAL4. The tissues were stained with an anti-GFP antibody for fru-GAL4 (green) and counterstained with nc82. Scale bar: 50 μm. (TIF) [file pgen.1008309.s003.tif]
