# Supplementary material for: Calmodulin-binding transcription factor shapes the male courtship song in Drosophila
Source: PLoS Genet. 2019 Jul 25;15(7):e1008309. doi: 10.1371/journal.pgen.1008309 (PMC6690551; doi:10.1371/journal.pgen.1008309)
Supplement: S1 Table — (DOCX) [file pgen.1008309.s004.docx]

| **S1 Table.** Genotypes of flies used in this study. | |
| --- | --- |
| **Fig. 1** | |
| A | *w^1118^/Y* |
|  | *w^1118^/Y; cro/cro* |
| B | *w^1118^/Y* |
| C | *w^1118^/Y; cro/cro* |
| D | *w^1118^/Y; cro/cro; BAC-CH321-22B08/+* |
| E | *w^1118^/Y* |
|  | *w^1118^/Y; cro/cro* |
|  | *w^1118^/Y; cro/cro; BAC-CH321-22B08/+* |
| F | *w^1118^/Y; cro/cro* |
| G | *w^1118^/Y* |
|  | *w^1118^/Y; cro/cro* |
|  | *w^1118^/Y; cro/+* |
|  | *w^1118^/Y; Df(2R)Np5/cro* |
|  | *w^1118^/Y; Df(2R)w45-30n/cro* |
|  | [*w^1118^/Y;*](http://flybase.org/search/symbol/FBgn/w)*Df(2R)*[*ED1791*](http://flybase.org/search/symbol/FBti/P%7B3%27.RS5%2B3.3%27%7DED1791)*/cro* |
|  | *w^1118^/Y; Df(2R)BSC408/cro* |
|  | *w^1118^/Y; Df(2R)w73-2/cro* |
|  | *w^1118^/Y; cro/cro; BAC-CH321-22B08/+* |
| H | *w^1118^/Y; Df(2R)Np5/cro* |
|  | *w^1118^/Y; Df(2R)w45-30n/cro* |
|  | [*w^1118^/Y;*](http://flybase.org/search/symbol/FBgn/w)*Df(2R)*[*ED1791*](http://flybase.org/search/symbol/FBti/P%7B3%27.RS5%2B3.3%27%7DED1791)*/cro* |
|  | *w^1118^/Y; Df(2R)BSC408/cro* |
|  | *w^1118^/Y; Df(2R)w73-2/cro* |
| **Fig. 2** | |
| A | *w^1118^/Y; cro/cro* |
| B | *w^1118^/Y; cro/cro* |
| C | *w^1118^/Y* |
|  | *w^1118^/Y; Mi{MIC}Camta[MI01132A]/cro* |
|  | *w^1118^/Y; Mi{MIC}Camta[MI04570]/cro* |
|  | *w^1118^/Y; cro/cro* |
|  | *w^1118^/Y; Mi{MIC}Camta[MI01580]/cro* |
|  | *w^1118^/Y; Mi{ET1}Camta[MB05101]/cro* |
|  | *w^1118^/Y; PBac{WH}Camta[f05489]/cro* |
| D | *w^1118^/Y* |
|  | *w^1118^/Y; Df(2R)BSC408/cro* |
|  | *w^1118^/Y; cro/cro* |
|  | *w^1118^/Y; Mi{MIC}Camta[MI04570]/cro* |
| **Fig. 3** | |
| A | *w^1118^/Y; cro-GAL4/cro* |
| B | *w^1118^/Y* |
|  | *w^1118^/Y; cro-GAL4/+* |
|  | *w^1118^/Y; cro-GAL4/cro* |
|  | *w^1118^/Y; cro-GAL4/cro; UAS-Camta^+^/+* |
| C | *w^1118^/Y; cro-GAL4/+* |
| D | *w^1118^/Y; cro-GAL4/cro* |
| E | *w^1118^/Y; cro-GAL4/cro; UAS-Camta^+^/+* |
| F | *w^1118^/Y* |
|  | *w^1118^/Y; cro-GAL4/+* |
|  | *w^1118^/Y; cro-GAL4/cro* |
|  | *w^1118^/Y; cro-GAL4/cro; UAS-Camta^+^/+* |
| **Fig. 4** | |
|  | *w^1118^/Y* |
|  | *w^1118^/Y; cro-GAL4/cro* |
|  | *w^1118^/Y; cro-GAL4/cro; UAS-Camta^+^/+* |
| **Fig. 5** | |
| A | *w/Y; +; UAS-Camta RNAi/+* |
|  | *w/Y; cro-GAL4/+* |
|  | *w/Y; cro-GAL4/+; UAS-Camta RNAi/+* |
| B | *w/Y; +; UAS-Camta RNAi/+* |
|  | *elav^GAL4^, w^1118^/Y; +; UAS-Camta RNAi/+* |
|  | *w/Y; +; UAS-Camta RNAi/nSyb^GAL4^* |
|  | *w/Y; UAS-Dcr2/+; repo^GAL4^/ UAS-Camta RNAi* |
| C | *yv/Y; +; UAS-Camta RNAi/+* |
|  | *yv/Y; +; UAS-Camta RNAi/Act5c^GAL4^* |
|  | *yv/Y; cro-GAL4/+; UAS-Camta RNAi/+* |
| D-H | *w/Y; cro-GAL4/+; UAS-mCD8::GFP/+* |
| **Fig. 6** | |
| B | *Canton-S* |
| C | *w/Y; cro-GAL4/cro* |
| D-F | *w/Y; cro-GAL4/+; UAS-mCD8::GFP/+* |
| **Fig. 7** | |
| A | *w/Y; +; UAS-Camta RNAi/+* |
|  | *w/Y; +; fru^GAL4^/+* |
|  | *w/Y; UAS-Dcr2/+; dsx^GAL4^/+* |
|  | *w/Y; +; fru^GAL4^/UAS-Camta RNAi* |
|  | *w/Y; UAS-Dcr2/+; dsx^GAL4^/UAS-Camta RNAi* |
| B | *w/Y; +; UAS-Camta RNAi/+* |
|  | *w/Y; Otd^FLP^, tubP>GAL80>/+; UAS-Camta RNAi/fru^GAL4^* |
|  | *w/Y; Otd^FLP^, tubP>GAL80>/+; UAS-Camta RNAi/dsx^GAL4^* |
|  | *w/Y; Otd^FLP^, tubP>stop>GAL80/+;UAS-Camta RNAi/fru^GAL4^* |
|  | *w/Y; Otd^FLP^, tubP>stop>GAL80/+;UAS-Camta RNAi/dsx^GAL4^* |
| C | *w/Y; +; UAS-Camta RNAi/+* |
|  | *tubP>GAL80>/Y; NP2631-GAL4/+; fru^FLP^/+* |
|  | *tubP>GAL80>/Y; NP2631-GAL4/+; fru^FLP^/UAS-Camta RNAi* |
|  | *w/Y; +; VT40556-GAL4/UAS-Camta RNAi* |
|  | *tubP>GAL80>/Y; p52a-GAL4/+; fru^FLP^/+* |
|  | *tubP>GAL80>/Y; p52a-GAL4/+; fru^FLP^/UAS-Camta RNAi* |
| D | *w/Y; UAS>STOP>mCD8::GFP/p52a-GAL4; fru^FLP^/+* |
| E-H | *w/Y; cro-GAL4/UAS>stop>mCD8::GFP; fru^FLP^/+* |
| **S2 Fig.** | |
| A-E | *w/Y; cro-GAL4/+; UAS-mCD8::GFP/+* |
| F-K | *w/Y; cro-GAL4/UAS>stop>mCD8::GFP; fru^FLP^/+* |
| **S3 Fig.** | |
| A | *w/Y; Otd^FLP^, tubP>GAL80>/UAS-mCD8::GFP; fru^GAL4^/+* |
| B | *w/Y; UAS-mCD8::GFP/+; fru^GAL4^/+* |
